# Case Report: Systemic amyloidosis unmasked by progressive hepatomegaly after splenectomy for non-traumatic spleen rupture in a patient with chronic liver disease

**DOI:** 10.3389/fmed.2026.1807002

**Published:** 2026-04-22

**Authors:** Li-Ping Sheng, Ya-Yun Zhang, Ming Zhang, Bo-Zhi Lin, Rui-Fang Ma, Li-Na Han, Hui Liu, Feng-Qin Hou, Gui-Qiang Wang

**Affiliations:** 1 Department of Infectious Diseases and Center for Liver Diseases, Peking University International Hospital, Beijing, China; 2 Department of Hematology, Peking University International Hospital, Beijing, China; 3 Department of Pathology, Peking University International Hospital, Beijing, China; 4 Department of Laboratory Medicine, Peking University International Hospital, Beijing, China; 5 Department of Pathology, Beijing Youan Hospital, Capital Medical University, Beijing, China; 6 Department of Infectious Diseases, Peking University First Hospital, Beijing, China

**Keywords:** amyloidosis, daratumumab, hepatomegaly, missed diagnosis, splenic rupture

## Abstract

**Background:**

Non-traumatic splenic rupture (NSR) is a rare manifestation of systemic amyloid light-chain amyloidosis (AL amyloidosis), a plasma cell dyscrasia best known for its cardiorenal involvement. This case is unique because the diagnostic clue from NSR was initially overlooked until the underlying disease unfolded into a multisystem disorder. The diagnostic clues were further obscured by the presence of chronic hepatitis B (HBV) and primary biliary cholangitis (PBC). The pitfalls and challenges, as well as the pathogenic link between AL amyloidosis and coexisting conditions, i.e., chronic HBV infection and PBC, are discussed.

**Case summary:**

A 46-year-old woman presented initially with non-specific upper gastrointestinal symptoms. Initial workup established the diagnosis of chronic HBV infection and PBC, but the disease progressed despite etiological therapy. NSR occurred 4 months later, and splenectomy pathology was inconclusive. The disease progressed, manifesting as cardiac dysfunction, acute kidney injury, subnephrotic-range proteinuria, and hepatomegaly. Serum and urinary-free light-chain levels and ratios were abnormal. Cardiac MRI suggested myocardial hypertrophy. Biopsy of the liver, bone marrow, and re-examination of the splenic specimen showed extensive amyloid deposition. She was diagnosed with multisystem AL amyloidosis (Mayo 2012 Stage III) and started on daratumumab, leading to significant clinical improvement.

**Conclusion:**

This case highlights the risk of “premature” attribution of manifestations of rare diseases to common comorbidities. While NSR should always be considered a sentinel event for systemic amyloidosis, the diagnostic clues can be easily overlooked in complex patients with overlapping diagnoses. A high index of suspicion and targeted histological evaluation of splenic tissue are imperative to avoid diagnostic delay and enable earlier treatment initiation for this serious disease.

## 1 Introduction

Amyloid light-chain amyloidosis (AL amyloidosis) is the most common form of systemic amyloidosis (1), a rare syndrome characterized by multi-organ deposition of misfolded immunoglobulin light-chain fibrils, leading to progressive organ dysfunction. While dyspnea and fatigue resulting from cardiac involvement are among the leading initial presenting symptoms of AL amyloidosis (2, 3), they are non-specific, contributing to diagnostic delay and poor prognosis (4, 5). Similarly, hepatic involvement typically manifests as hepatomegaly and a cholestatic pattern of liver chemistry abnormalities (6) and is common, being diagnosed radiologically in 50% (7) and pathologically in 70% of AL amyloidosis cases (8).

Paradoxically, non-traumatic splenic rupture (NSR), an exceedingly rare and life-threatening initial manifestation of systemic amyloidosis, often serves as the key diagnostic clue in many well-documented case reports (9). However, since NSR can also be predisposed to by congestive splenomegaly secondary to portal hypertension in chronic liver diseases (10, 11), its diagnostic specificity for an infiltrative disorder in this setting can be compromised.

Here, we present an instructive case where systemic AL amyloidosis revealed itself in a stuttering manner. The diagnosis was initially missed at the time of NSR and was only unmasked 6 months later, prompted by hepatomegaly and multi-organ dysfunction, and finally confirmed by biopsy of the enlarged liver and re-examination of the previously dissected spleen. This convoluted disease course highlights the importance of maintaining sustained vigilance for major unexplained clinical events. In this case, the salient lesson is that etiologies such as amyloidosis should be thoroughly investigated in the presence of NSR, especially in the setting of chronic hepatitis B (HBV) and primary biliary cholangitis (PBC), to avoid diagnostic delay and its potential consequences.

## 2 Case presentation

A 46-year-old woman with an unremarkable medical history developed nausea, vomiting, fatigue, and anorexia 10 months before admission to our hospital. She did not smoke or drink and denied any history of trauma or medical/surgical procedures. She did not seek medical help until 4 months later (6 months before admission), when her symptoms became more severe despite self-administered Chinese herbal medicine.

Laboratory studies revealed a mild elevation in aspartate aminotransferase (AST). Serological testing was significant for positive HBsAg, HBeAb, and HBcAb, with a low HBV DNA level (80 IU/mL). Other abnormal findings included a low-titer (1:100) antinuclear antibody (ANA) and positive anti-sp100 and anti-promyelocytic leukemia (PML) protein antibodies. Abdominal ultrasound showed gallbladder wall thickening and splenomegaly (165 mm in length), without evidence of parenchymal or vascular abnormality of the liver or ascites.

Upper endoscopy revealed fungal esophagitis and chronic atrophic gastritis. Chronic hepatitis B virus (HBV) infection was diagnosed, and tenofovir alafenamide (TAF) was prescribed. With little symptomatic improvement, laboratory tests were repeated 2 weeks later (5.5 months before admission), showing alkaline phosphatase (ALP) at 328 U/L and gamma-glutamyl transferase (GGT) at 258 U/L. Abdominal CT showed a normal liver size and contour. Ursodeoxycholic acid was added empirically.

Five months before admission, the patient experienced an abrupt onset of abdominal pain. In the emergency department of another hospital, splenic rupture was diagnosed, and an emergency splenectomy was performed. Initial examination of the resected spleen described an enlarged (15.5 × 9 × 7 cm), lacerated spleen with subcapsular hemorrhage grossly, and microscopically showed fibrosis and inflammatory cell infiltration. The etiology of the rupture remained unexplored.

The patient’s initial complaint continued over the subsequent months. Two weeks before admission to our center, she presented to a local hospital with severe fatigue, exertional dyspnea, and hypotension. Critical laboratory findings at that time included severe hyponatremia (115 mmol/L), hypoalbuminemia (22.5 g/L), acute kidney injury (creatinine 114 μmol/L), and subnephrotic-range proteinuria (2.2 g/24 h).

Abdominal ultrasound revealed marked hepatomegaly (right lobe oblique diameter 14.6 cm, left lobe anteroposterior diameter 8.0 cm) with coarse parenchymal echotexture and moderate ascites. Decompensated cirrhosis was presumed, and therapy, including albumin infusion and norepinephrine support, was instituted, with limited clinical improvement. She was then referred to our department for further evaluation.

On admission, the patient was ill-appearing but alert. Vital signs included a temperature of 37.0 °C, a heart rate of 88 beats per minute, a respiratory rate of 18 breaths per minute, and a blood pressure of 84/52 mm Hg while receiving intravenous norepinephrine continuously at a dose of 0.5 μg/kg/min.

Abdominal examination revealed a palpable liver edge 3 cm below the right costal margin and shifting dullness. A well-healed surgical scar was noted in the left upper quadrant. Cardiac examination revealed no murmurs, but jugular venous pressure was elevated. There was mild symmetrical pitting edema in the lower extremities.

Laboratory findings on admission are summarized in Table 1. Notable results included elevated cardiac biomarkers [troponin T 101.4 ng/L, N-terminal pro-B-type natriuretic peptide (NT-proBNP) 9,818 pg/mL], coagulopathy [international normalized ratio (INR) 1.68], and mildly elevated serum creatinine (92 μmol/L) and urea (9.85 mmol/L).

**Table 1:** Laboratory results.

Laboratory	Reference range	6 months before time zero	5.5 months before time zero	On admission (time zero)	After two cycles of chemotherapy (6 weeks after time zero)
Complete blood count					
Leukocytes (×10^9^/L)	3.5–9.5	8.2		9.6	8.9
Hematocrit (%)	35–45	34		30	23.4
Hemoglobin (g/dL)	115–150	110		105	78
Platelets (×10^9^/L)	125–350	201		151	93
CRP (mg/L)	<10	4		5	–
ESR (mm/1st h)	0–20	–		6	–
Ferritin (ng/mL)	4.6–204	–		131	–
Cardiac biomarkers					
NT-proBNP (pg/mL)	<125	–		9,818	5,244
cTnT (ng/L)	<14	–		101	25
CK-MB (U/L)	<5	–		13	3
Myo (ng/mL)	25–58	–		107	51
Chemistry					
Scr (μmol/L)	45–84	81		92	124
BUN (mmol/L)	2.6–7.5	6		9.8	14.9
ALT (U/L)	7–40	38		28	13
AST (U/L)	13–35	66		72	43
GGT (U/L)	7–45	–	258	85	95
ALP (U/L)	35–100	–	328	230	148
TBil (μmol/L)	3.4–23.3	21		22	45
LDH (U/L)	120–250	–		407	270
Coagulation					
D-dimer (ng/mL)	<250	–		6,983	8,668
Prothrombin time (s)	9.4–12.5	–		18.5	16.1
Fibrinogen (mg/dL)	200–400	–		149	145
International normalized ratio	0.9–1.2	–		1.68	1.35
PTA (%)	70–120	–		49	58
HIV (C.O.I)	<1	Negative		0.2	–
HBsAg (IU/mL)	<0.03	Positive		719	–
HBeAb (C.O.I)	<50	Positive		99	–
HBcAb (C.O.I)	<0.99	Positive		274	–
HBeAg (C.O.I)	<1	Negative		0	–
HBsAb (mIU/mL)	≤10	Negative		0.9	–
HCV (C.O.I)	<1	Negative		0	–
CMV-DNA (copies/mL)	<400	Negative		Negative	–
EBV-DNA (copies/mL)	<400	Negative		Negative	–
24-h urine protein (g/L)	28–141	2.2		2.8	–
ANA (titer)	<1:40	1:100		Negative	–
AMA (titer)	<1:40	Negative		Negative	–
AMA-M2 (RU/mL)	0–20	Negative		3.2	–
Anti-SP100	–	+++		NA	–
IgM (g/L)	0.4–2.3	1.2		1.1	–
IgG (g/L)	7–16	6		5.9	–
Urinary kappa chains (mg/L)	0–7.9	–		101	–
Urinary lambda chains (mg/L)	0–4.3	–		24	–
Serum free light chains					–
Free kappa (mg/L)	3.3–19.4	–		194	–
Free lambda (mg/L)	5.7–26.1	–		24	–
Kappa/lambda ratio	0.26–1.95	–		7.8	–
HBVDNA (IU/mL)	<10	80		<10	–

CRP, C-reactive protein; ESR, erythrocyte sedimentation rate; NT-proBNP, N-terminal pro-B-type natriuretic peptide; cTnT, cardiac troponin T; CK-MB, creatine kinase-MB; Myo, myoglobin; Scr, serum creatinine; BUN, blood urea nitrogen; ALT, alanine aminotransferase; AST, aspartate aminotransferase; GGT, gamma-glutamyl transferase; ALP, alkaline phosphatase; TBil, total bilirubin; LDH, lactate dehydrogenase; PTA, prothrombin activity; HBsAg, hepatitis B surface antigen; HBeAb, hepatitis B e antibody; HBcAb, hepatitis B core antibody; HBeAg, hepatitis B e antigen; HBsAb, hepatitis B surface antibody; HCV, hepatitis C virus; CMV, cytomegalovirus; EBV, Epstein–Barr virus; ANA, antinuclear antibody; AMA, anti-mitochondrial antibody; AMA-M2, anti-mitochondrial M2 antibody; IgM, immunoglobulin M; IgG, immunoglobulin G; HBV DNA, hepatitis B virus DNA.

**Figure 1:**
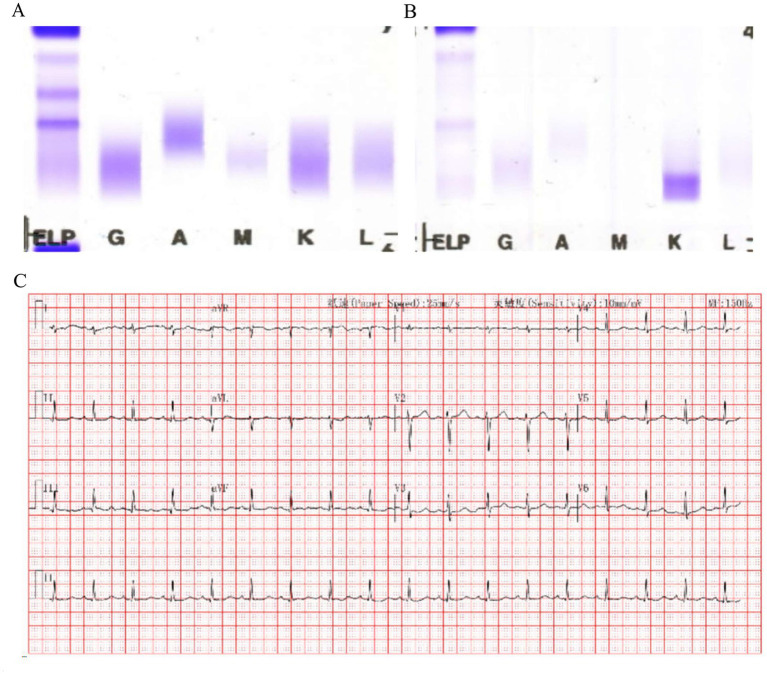
Serum and urinary protein electrophoresis, immunofixation electrophoresis, and electrocardiogram. **(A)** Serum protein electrophoresis and immunofixation electrophoresis did not detect a significant monoclonal band. **(B)** Urine immunofixation electrophoresis revealed the presence of a monoclonal band in the kappa light-chain region. **(C)** Electrocardiogram on admission showed sinus tachycardia and non-specific ST–T changes.

Serum protein electrophoresis (SPEP) and immunofixation (SIFE) revealed no evidence of monoclonal protein (Figure 1A). Urine immunofixation electrophoresis (IFE) detected a dense free light chain (FLC) kappa monoclonal band (Figure 1B).

Serum immunoglobulin-free light chain (sFLC) assay (The Binding Site Group, Birmingham, UK) quantified kappa FLC at 194 mg/L [reference range (ref): 3.3–19.4 mg/L], lambda FLC at 24 mg/L (ref: 5.7–26.1 mg/L), a kappa-to-lambda ratio of 8.1 (ref: 0.26–1.95), and a kappa–lambda difference of 170 mg/L.

**Figure 2:**
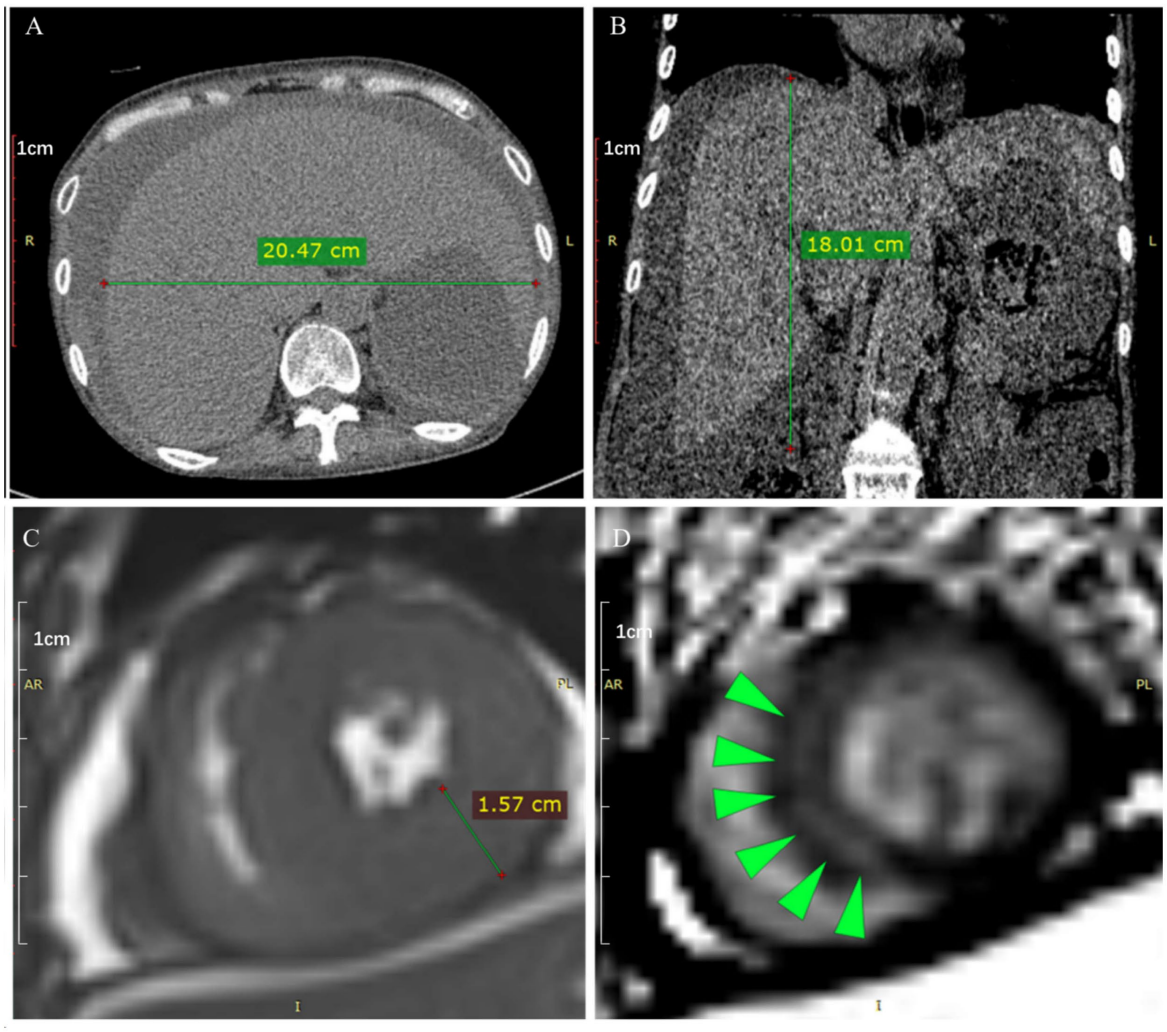
Non-contrast abdominal computed tomography (CT) scan and cardiac magnetic resonance imaging (MRI). Measurements of the transverse and craniocaudal lengths of the liver at the axial **(A)** and coronal **(B)** sections are shown. Hypertrophic myocardium, as indicated by a wall thickness of 1.57 cm at the end-systolic phase **(C)**, and left ventricular subendocardial enhancement **(D)** are highlighted (green arrowheads) by phase-sensitive inversion recovery (PSIR) late gadolinium enhancement (LGE) imaging, both in the short-axis plane.

Electrocardiogram (ECG) on admission revealed sinus tachycardia with non-specific ST–T wave changes in the precordial leads (Figure 1C). Non-contrast abdominal CT scan revealed marked hepatomegaly, i.e., enlargement of both hepatic lobes (Figures 2A,B), and ascites.

Echocardiography revealed left ventricular wall thickening with preserved ejection fraction (63%). Cardiac magnetic resonance imaging (MRI) showed biventricular wall thickening (Figure 2C) with delayed gadolinium enhancement of the myocardium, interatrial septum, and valves, suggestive of cardiac amyloid infiltration (Figure 2D).

**Figure 3:**
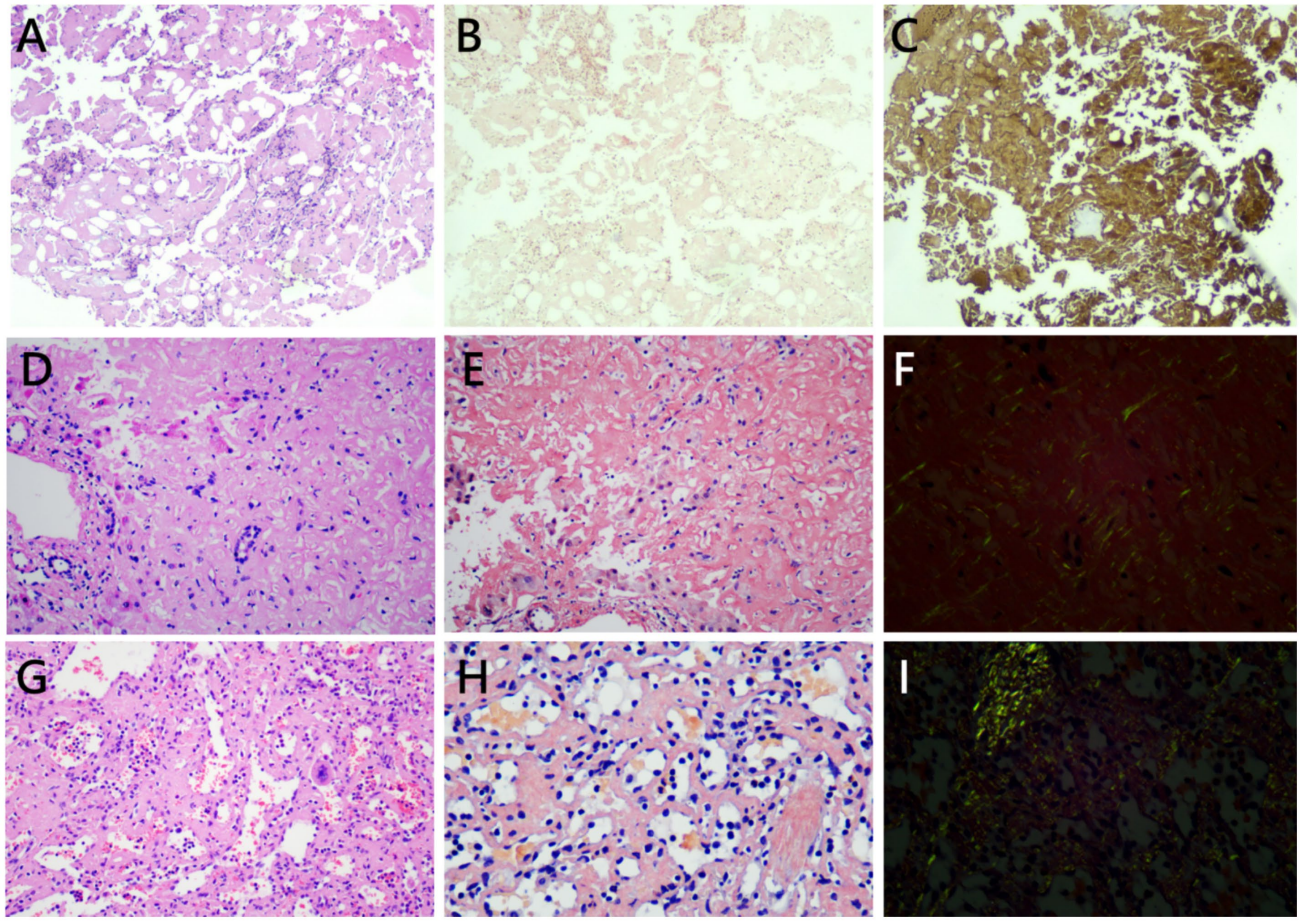
Examination of biopsied bone marrow **(A–C)**, liver **(D–F)**, and spleen **(G**–**I)**. Amorphous eosinophilic material was diffusely deposited in all depicted tissues under H&E stain (**A,D,G**, ×200 magnification), with a red-orange appearance under Congo red stain on light microscopy (**B,E,** ×200 magnification; **H**, ×400 magnification) and apple-green birefringence under polarized light **(F,I)**. Immunoglobulin light chain *λ* stain **(C)** of the bone marrow is also shown. The structures of the portal tracts and lobules were severely distorted by heavy amyloid deposition, and hepatocytes were diffusely destroyed **(D,E)**. The portal tract was infiltrated by lymphocytes with biliary injury **(D)**. H&E, hematoxylin and eosin.

Bone marrow aspirate cytology revealed a plasma cell percentage of 6%. Biopsy (Figure 3A) showed scant hematopoietic cells. Critically, interstitial and vascular deposits of amorphous eosinophilic material were observed. Immunohistochemistry of the bone marrow specimen showed that these deposits appeared red-orange on Congo red staining (Figure 3B) and were positive for both kappa and lambda light chains, with lambda predominance (Figure 3C), consistent with marrow involvement by light chain amyloidosis. Plasma cells were marginally increased but did not show definitive clonality by immunohistochemistry.

A percutaneous liver biopsy was performed. Histopathological examination showed extensive deposition of amorphous eosinophilic material within the sinusoids, leading to compression and atrophy of the hepatocyte plate (Figure 3D). The material stained positive with Congo red (Figure 3E) and exhibited apple-green birefringence under polarized light (Figure 3F). The initial spleen specimen was also retrieved and reviewed. Re-examination revealed amyloid deposition in the splenic cords and vessel walls (Figures 3G–I), confirming that the non-traumatic rupture was indeed secondary to splenic amyloidosis.

Based on the constellation of an abnormal serum free light-chain ratio, characteristic cardiac MRI findings, significant renal involvement (subnephrotic-range proteinuria and renal impairment), and histological demonstration of AL in the liver, spleen, and bone marrow, the diagnosis of systemic AL (lambda) amyloidosis (Mayo 2012 Stage III) with multisystem involvement (hepatic, splenic, cardiac, renal, and bone marrow) was established.

**Figure 4:**
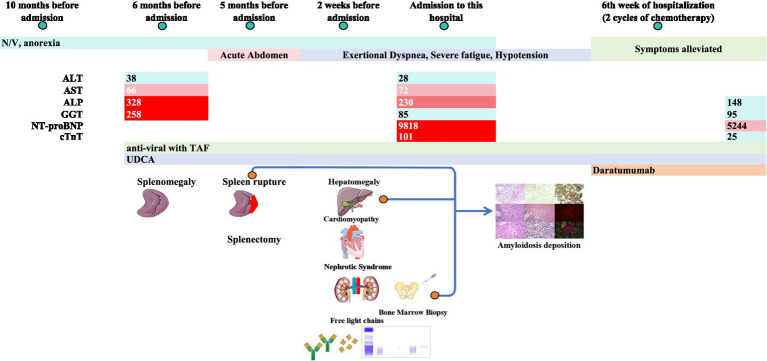
Timeline of the disease course with major events, including symptoms, selected laboratory results, and key diagnostic testing results. Alanine aminotransferase (ALT), aspartate aminotransferase (AST), alkaline phosphatase (ALP), gamma-glutamyl transferase (GGT), N-terminal pro-B-type natriuretic peptide (NT-proBNP), cardiac troponin T (cTnT), and nausea and vomiting (N/V). Created with illustrations from Servier Medical Art (https://smart.servier.com), licensed under CC BY 4.0 (https://creativecommons.org/licenses/by/4.0/).

The patient was then transferred to the hematology department. Based on recommendations for first-line therapy for newly diagnosed AL amyloidosis (10), weekly infusion of daratumumab 600 mg was initiated. After two cycles, her exercise tolerance and overall clinical status improved significantly, allowing her to be discharged with scheduled outpatient therapy (Figure 4).

## 3 Discussion

The case reflects the diagnostic challenge associated with AL amyloidosis, which often requires a median of 2.5 years (12) or consolation with 3–4 physicians (2) before a correct diagnosis is reached. From a retrospective perspective, there were several critical time points in our case when the suspicion of systemic amyloidosis could have been raised.

First, when the patient presented with 4 months of non-resolving upper gastrointestinal complaints, the etiology of isolated splenomegaly was undetermined. Underlying causes of splenomegaly should be sought, mainly including hematological, infectious, and congestive diseases (13, 14).

Although diagnostic workup then established chronic HBV infection, in the absence of evidence of cirrhosis or clinically significant portal hypertension (CSPH) (15), it is very unlikely for splenomegaly to occur at this stage of liver disease. A weak association between HBV infection and the risk of developing multiple myeloma has previously been reported (16). While cases of systemic amyloidosis in patients with HBV were very rare, with only one reported case involving HIV co-infection, where HBV was postulated to act as a chronic immunological stimulator (17), a causal relationship between HBV and AL amyloidosis cannot be established thus far. In contrast, the risk of HBV reactivation during chemotherapy is more well established (18) and should be cautiously managed with antiviral therapy (19).

Since anti-sp100 and anti-PML antibodies have high specificity for primary biliary cholangitis (PBC) diagnosis (20) and prognosis (21), it is reasonable to lower the threshold for a proactive liver biopsy to clarify competing etiologies, particularly in the setting of normal ALP (22). Less invasive diagnostic procedures, such as bone marrow or fat pad biopsy and aspiration, can be considered.

Secondly, when the patient developed biochemical cholestasis shortly thereafter, a presumptive diagnosis of PBC was established. Liver biopsy was still warranted at this time to rule out other conditions (23), given the rapid onset of cholestasis. Longstanding PBC has been reported as a potential precipitant only for amyloid A (AA) amyloidosis (24, 25). In our case, PBC appeared to be in its early course. It is plausible that PBC was a consequence of the underlying plasma cell dyscrasia, given evidence supporting the role of plasma cells in PBC pathogenesis (26).

Third, the next major event of spontaneous splenic rupture offered an unprecedented, though costly, opportunity to detect amyloidosis. The original histopathology, based on routine hematoxylin and eosin (H&E) staining, described the findings as “fibrosis.” This underscores a diagnostic pitfall in practice: although fibrosis and amyloidosis can often be differentiated unequivocally based on morphology alone (27), misinterpretation can occur, requiring special staining (e.g., Masson’s trichrome and Congo red stains) (28). While the H&E stain may show extracellular, amorphous eosinophilic material (29), it lacks the specificity of Congo red stain for a definitive diagnosis (30).

Subsequent occurrence of exertional dyspnea, circulatory collapse, hepatomegaly, and non-diabetic subnephrotic-range proteinuria reflected the progression of a previously unrecognized underlying systemic disease to its full-blown stage. Several clinical “red flags” prompted evaluation of systemic amyloidosis, serving as key teaching points in this case:

First, while acute decompensation of cirrhosis and resuscitation-associated fluid overload seemed to explain the overall picture, the clinical deterioration could not be fully explained by chronic liver disease. The patient had a normal baseline platelet level, and endoscopy confirmed compensated chronic liver disease. The risk of decompensation in this setting is very low, probably less than 0.5 per 1,000 person-years, based on a large prospective Chinese cohort of HBV-related cirrhosis (31).

Second, the prominent feature of myocardial hypertrophy in the absence of a history of hypertension, combined with organomegaly, subnephrotic-range proteinuria, and abnormal serum free light chain (sFLC) levels, strongly suggested an infiltrative process.

These prominent features necessitate thorough evaluation. Cardiac MRI can be very helpful in this setting. A characteristic diffuse subendocardial late gadolinium enhancement (LGE) pattern on MRI is highly sensitive and specific for cardiac amyloidosis (32). Given the abnormal sFLC, bone marrow biopsy is warranted to quantify plasma cell infiltration and definitively classify the amyloid deposition (33).

Unfortunately, by the time the diagnosis was established, profoundly elevated cardiac biomarkers indicated advanced disease, according to Mayo 2012 (Stage III) or European modification of the Mayo 2004 staging system (Stage IIIB). Prognosis is unfavorable, with a median survival of 14 and 7 months in each derivation cohort (5, 34), respectively.

This diagnostic delay is typical. According to a large claims database of AL amyloidosis, the median time from symptom onset to diagnosis was 32 months for patients with heart involvement (35). In another cohort, 76% of patients already had Mayo 2004 Stage III disease at diagnosis (36). However, non-traumatic spleen rupture (NSR), a rare manifestation of AL amyloidosis (9), almost always leads to immediate histopathological diagnosis, but was missed in our case.

Documented obstacles in this context include referral to a dedicated amyloid medical center for diagnosis confirmation in one case, associated with a 4-month lapse after emergent splenectomy (37). Another case described a 12-month gap between NSR and amyloidosis diagnosis, but details were lacking (38). In our case, failure to recognize NSR as a sentinel event for the underlying systemic disorder should be considered the main contributor to the diagnostic delay. Chronic HBV infection and PBC further complicated the situation and introduced additional barriers to early recognition of the true culprit. As such, pivotal diagnostic clues, as already mentioned, may go unnoticed despite being revealed.

In summary, we present a rare case that exemplifies the well-documented association between non-traumatic spleen rupture and immunoglobulin light-chain systemic amyloidosis. We also candidly discussed the pitfalls in the complex diagnostic process, illustrating how clinical insights can be obscured by the presence of HBV and PBC. This case serves as a reminder of the practical challenges of rare diseases faced by both clinicians and patients.

## Data Availability

The original contributions presented in the study are included in the article/supplementary material, further inquiries can be directed to the corresponding authors.
